# A Comprehensive Analysis of the Impact of Nutrient Intakes on the Stages and Mortality of Cardiovascular‐Kidney‐Metabolic Syndrome

**DOI:** 10.1002/fsn3.71747

**Published:** 2026-04-13

**Authors:** Jianbo Qing, Jiaying Hu, Kaili Qin, Mingjing Gao, Yimiao Ma, Sayna Norouzi, Junnan Wu

**Affiliations:** ^1^ Department of Nephrology, Sir Run Run Shaw Hospital Zhejiang University School of Medicine Hangzhou China; ^2^ Department of Nephrology Shanxi Provincial People's Hospital (Fifth Hospital) of Shanxi Medical University Taiyuan China; ^3^ Division of Nephrology, Department of Medicine Loma Linda University Medical Center Loma Linda California USA

**Keywords:** diet, global disease burden (GBD), predictive model, restricted cubic spline, weighted regression model

## Abstract

Cardiovascular–kidney–metabolic (CKM) syndrome remains highly prevalent worldwide and is associated with substantial mortality. As diet is a key modifiable determinant in the development and progression of CKM syndrome, this study aimed to investigate the associations between dietary nutrient intake and CKM syndrome to inform prevention and management strategies. We integrated data from the Global Burden of Disease (GBD) study and the National Health and Nutrition Examination Survey (NHANES) to provide both global and individual‐level insights. Global age‐standardized disability‐adjusted life years (ASDR) and age‐standardized mortality rates (ASMR) attributable to dietary risks were analyzed for six CKM‐related diseases. Multinomial logistic regression and weighted Poisson regression were used to assess associations between 26 dietary nutrients and CKM syndrome stages and mortality, and a nutrient‐based mortality risk prediction model and nomogram were further developed and validated. Marked regional variations in ASDR and ASMR were observed, particularly in Eastern Europe. Significant differences in nutrient intake patterns were found across CKM syndrome stages. Lower potassium and higher sodium intake were associated with advanced CKM stages. Cholesterol intake was associated with increased all‐cause mortality risk, whereas higher intakes of dietary fiber, choline, vitamin K, and PFA 20:4 were associated with reduced mortality risk. The nutrient‐based nomogram demonstrated good predictive performance for mortality risk in CKM syndrome. In conclusion, this study integrates global burden estimates with individual‐level nutrient data to characterize dietary determinants of CKM syndrome progression and mortality, highlighting modifiable nutrient targets and providing a data‐driven framework for dietary risk prediction.

## Graphical abstract

Higher sodium and lower potassium intake were linked to advanced CKM syndrome stages, while specific nutrients influenced mortality risk and were incorporated into a predictive nomogram.

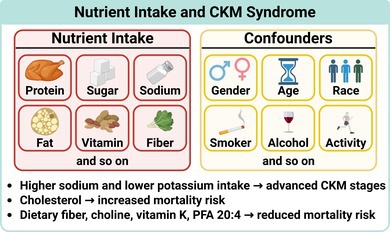

## 1 Introduction

With the global aging population and shifts in lifestyle, the incidence and mortality of chronic diseases have been steadily rising, particularly those related to cardiovascular, kidney, and metabolic disorders, such as diabetes and obesity (Ren et al. 2024). Due to their strong pathophysiological interconnections, the American Heart Association (AHA) introduced the cardiovascular–kidney–metabolic (CKM) syndrome framework to optimize disease prevention and management (Ndumele et al. 2023). Currently, over one billion people worldwide are affected by CKM syndrome, and in the United States, at least 15% of adults progress to advanced stages (stages 3 or 4), making it one of the leading causes of death among adults and placing an increasing burden on global health (Ferdinand 2024; Aggarwal et al. 2024).

The management of CKM syndrome primarily relies on pharmacological treatments and lifestyle interventions, with diet playing a particularly substantial role (Pena et al. 2025). As a core modifiable factor in the onset, progression, and prognosis of CKM syndrome, dietary interventions have shown significant benefits in managing cardiovascular diseases (CVD), diabetes mellitus (DM), and chronic kidney disease (CKD) (Shi et al. 2025; Hung et al. 2025; Pérez‐Torres et al. 2022). However, most existing studies have focused on single dietary components or specific food groups, often overlooking the complex interplay among multiple nutrients across CKM syndrome stages and their collective impact on disease progression and mortality. Moreover, few studies have integrated global disease burden assessments with individual‐level nutrient analyses, limiting our understanding of how specific nutrient patterns contribute to CKM syndrome development and outcomes.

**FIGURE 1:**
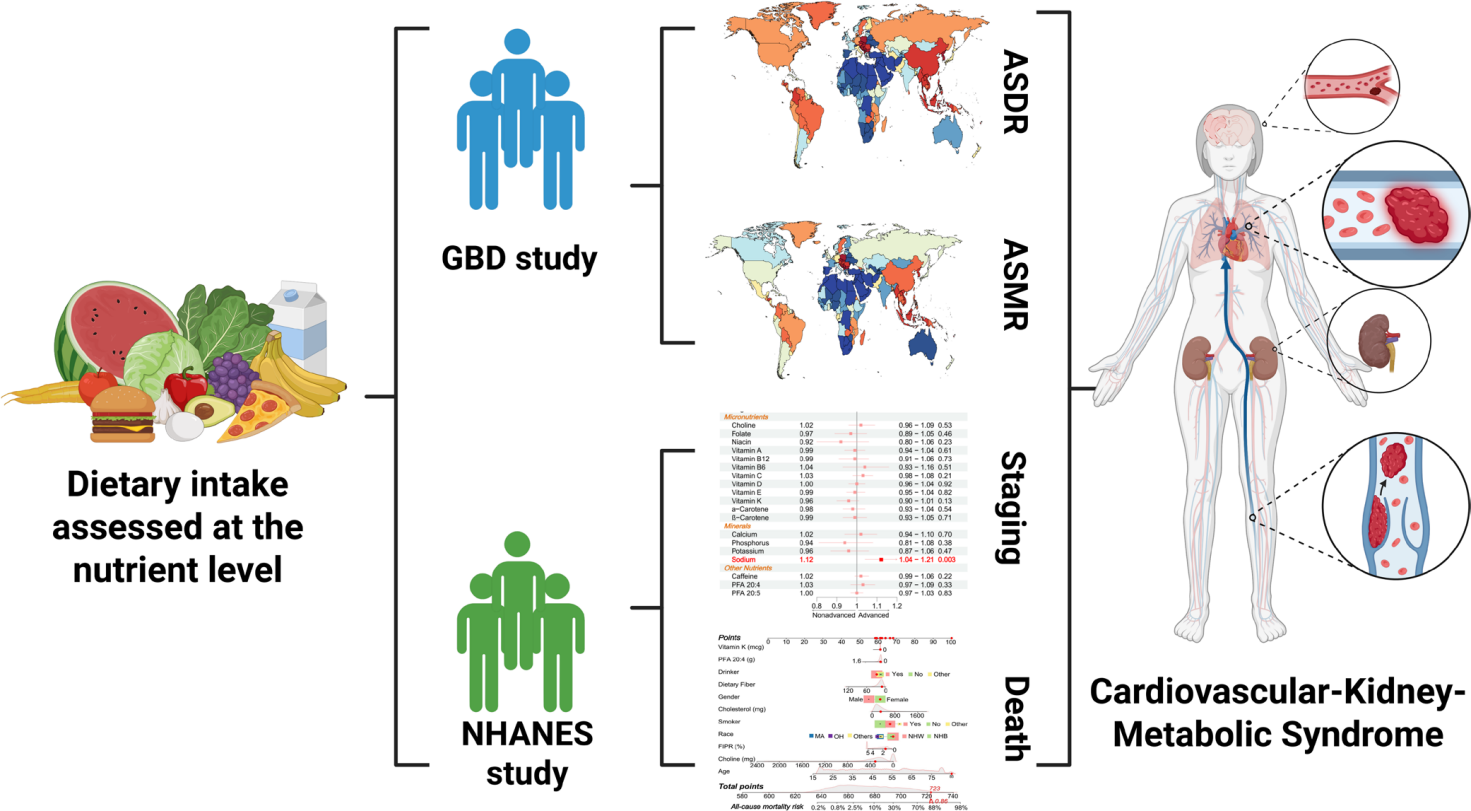
Overview of the study design. This study provides a comprehensive assessment of the relationship between nutrient intake and CKM syndrome by collecting data from the GBD and NHANES studies. Abbreviations: ASDR, age‐standardized disability‐adjusted life years; ASMR, age‐standardized mortality rate; GBD, Global Burden of Disease; NHANES, National Health and Nutrition Examination Survey.

To address these gaps, this study combines data from the Global Burden of Disease (GBD) project and the National Health and Nutrition Examination Survey (NHANES) to comprehensively evaluate the associations between dietary nutrient intake and CKM syndrome (Figure 1). By examining 26 nutrients in relation to CKM syndrome staging and all‐cause mortality, and by developing a nutrient‐based mortality risk prediction model, this study provides novel insights into the nutritional determinants of CKM syndrome and offers a new, data‐driven framework for targeted dietary prevention and management strategies.

## 2 Methods

### 2.1 Data Resources and Study Population

The GBD 2021 study assessed the global disease burden and exposure to 88 risk factors across 204 countries from 1990 to 2021. Using a comparative risk assessment framework, the study generated age‐standardized disability‐adjusted life years rates (ASDR) and age‐standardized mortality rates (ASMR), which were weighted according to the population's age distribution. These data are available at the official website (https://www.healthdata.org), from which we obtained ASDR and ASMR for diseases linked to dietary risk exposure across the 204 countries (Jeong et al. 2025).

The National Health and Nutrition Examination Survey (NHANES) data collected through a stratified, multistage sampling design, provides a cross‐sectional survey of the U.S. population, including demographic information, laboratory results, and questionnaire data, spanning from 1999 to 2023 (Aggarwal et al. 2024). Due to missing dietary interview data in 1999–2000 and high rates of missing serum creatinine data from 2021 to 2023, this study primarily used data from 2001 to 2020. Although CKM syndrome is more commonly diagnosed in adulthood, growing evidence suggests that its pathophysiological processes often originate earlier in life, with metabolic abnormalities such as obesity, dyslipidemia, and impaired glucose metabolism increasingly prevalent among children and adolescents. Because the GBD study reports age‐standardized rates across the entire population, restricting NHANES analyses to adults alone may underestimate the overall burden of CKM syndrome by failing to capture early‐life metabolic risk states. Therefore, we included both adult and pediatric participants from NHANES to better reflect a life‐course perspective of CKM syndrome development and to improve alignment with the population‐based age‐standardized framework used in the GBD study (Salama et al. 2023; Ng et al. 2024). Although the pediatric sample size was relatively small, its inclusion helps account for early metabolic dysregulation that may precede overt CKM syndrome in later life, and inclusion of children and adolescents was intended to capture early metabolic risk rather than clinical CKM syndrome diagnoses.

GBD analyses were conducted using aggregated, population‐level estimates that are publicly available from the GBD website rather than individual‐level data; therefore, no additional inclusion or exclusion criteria were applied to the GBD component. In contrast, individual‐level data from the NHANES were used.

Participant mortality data from 2001 onwards were obtained from the NHANES database, with the most recent data available up to 2018. This dataset includes information on whether participants were deceased and the cause of death. Deaths were considered CKM syndrome‐related if the primary cause was CVD, cerebrovascular disease, diabetes, or kidney diseases such as nephritis, nephrotic syndrome, or CKD.

This study follows the STROBE (Strengthening the Reporting of Observational Studies in Epidemiology) guidelines, utilizing de‐identified data, and as both the GBD and NHANES study protocols had prior ethical review approval, no additional ethical approval was required.

### 2.2 Definition and Staging of CKM Syndrome

Based on previous studies, we included six diseases in the GBD data analysis: atrial fibrillation and flutter (AFF), CKD, DM, ischemic heart disease (IHD), lower extremity peripheral arterial disease (LEPA), and stroke. In the GBD study, AFF was diagnosed based on electrocardiogram features such as irregular R‐R intervals, indicative of atrial fibrillation and flutter. CKD was defined by the presence of chronic kidney dysfunction, assessed through the albumin‐to‐creatinine ratio (ACR) and estimated glomerular filtration rate (eGFR). DM was diagnosed when fasting blood glucose levels were ≥ 7 mmol/L or when the individual was receiving treatment for blood glucose management (including insulin injections or oral medications). IHD was identified based on imaging, electrocardiogram, and myocardial injury markers, indicating restricted blood flow to the heart. LEPA was defined by an ankle‐brachial index (ABI) ≤ 0.9. Stroke was defined as an acute neurological dysfunction, excluding transient ischemic attacks (Xie et al. 2025).

The CKM syndrome staging for NHANES participants was based on the criteria established by Zhu et al. (2024), with classification into five stages (0–4). Stage 0 was defined by a BMI between 18.5 and 24.9, a waist circumference of < 102 cm for males and < 88 cm for females, and the absence of CKM syndrome risk factors. Stage 1 primarily includes individuals with overweight, defined as BMI ≥ 25, waist circumference ≥ 88 cm for males and ≥ 102 cm for females, as well as those in the prediabetic state or using antidiabetic medications. At Stage 2, participants were characterized by the presence of metabolic risk factors, including hypertension, diabetes, metabolic syndrome, fasting serum triglycerides ≥ 135 mg/dL, and moderate to high‐risk CKD per Kidney Disease Improving Global Outcomes (KDIGO) criteria. Stages 3 and 4 rely on the 10‐year CVD risk prediction, calculated using the PREVENT equations, which are applicable only to individuals aged 30–79 years. Stage 3 includes individuals with predicted high or very high 10‐year CVD risk or those with very‐high‐risk KDIGO CKD stages. Stage 4 encompasses individuals diagnosed with coronary artery disease, angina, myocardial infarction, heart failure, or stroke. Additionally, the participants were categorized into two groups based on CKM syndrome staging: advanced stages (stage 3–4) and non‐advanced stages (stage 0–2).

### 2.3 Nutrient Intake

Nutrient intake data were obtained through dietary questionnaires, with the average intake calculated over a two‐day period. We collected data on 26 nutrients, which were categorized into four groups: macronutrients, micronutrients, minerals, and other nutrients. The macronutrients included protein, carbohydrates, total sugars, dietary fiber, total fat, and saturated fatty acids. Micronutrients comprised total vitamin A, α‐carotene, β‐carotene, niacin, vitamin B6, total folate, vitamin B12, vitamin C, vitamin D, vitamin K, vitamin E, and total choline. Minerals included calcium, phosphorus, sodium, and potassium. Other nutrients included caffeine, PFA 20:4, and PFA 20:5. Participants with missing data for any of the listed nutrients were excluded from the analysis.

### 2.4 Confounding Factors

In selecting potential confounders, we relied on existing literature and a conceptual causal framework informed by directed acyclic graph (DAG) theory (Figure S1) (Digitale et al. 2022; Cortes et al. 2016). DAGs were constructed using the R packages dagitty (Version 0.3–4) and ggdag (Version 0.2.13) to represent assumed causal relationships and to guide confounder selection. We considered variables that could plausibly influence both dietary nutrient intake and CKM syndrome‐related outcomes.

Specifically, we adjusted for sociodemographic characteristics, including age (continuous), sex, race/ethnicity (Mexican American, Other Hispanic, Non‐Hispanic White, Non‐Hispanic Black, and other races), and socioeconomic status, assessed using the family income‐to‐poverty ratio (FIPR; continuous), as these factors are known to be associated with dietary behaviors and cardiometabolic risk (Artegoitia et al. 2021; Wang, Gao, et al. 2024).

Lifestyle‐related factors were also included, namely smoking status (defined as having smoked at least 100 cigarettes during one's lifetime), alcohol consumption (defined as consuming ≥ 12 alcoholic beverages per year), and physical activity (defined as engaging in ≥ 150 min of moderate‐intensity or ≥ 75 min of vigorous‐intensity activity per week). These variables were treated as confounders rather than mediators, as they are not considered to lie on the direct causal pathway between dietary nutrient intake and CKM syndrome, but may jointly influence both the exposure and the outcome (Jin et al. 2023; Zhang et al. 2024).

### 2.5 Statistical Analysis and Visualization

All analyses were conducted using R (4.3.0). Since NHANES data were collected based on complex sampling, Pearson's weighted *χ* ^2^ test was used for categorical variables to analyze differences across CKM syndrome stages, and weighted linear regression was applied to continuous variables. Weighted multinomial logistic regression models were used to assess the relationship between nutrient intake and CKM syndrome staging, with age, sex, race, FIPR, smoking, alcohol consumption, and physical activity adjusted as confounders. For participants with missing or refused responses on smoking, alcohol use, or physical activity, these were treated as missing data and included in the model as factors for adjustment. To evaluate the impact of nutrient intake on CKM syndrome progression and mortality risk, weighted modified Poisson regression models were used to compute relative risks (RR), adjusting for confounders. Due to the high variability and small fluctuations in the values of most nutrients, Z‐standardization was performed before conducting regression analysis.

As this study is exploratory and aimed at generating hypotheses for future research, multiple testing corrections were not performed to avoid type I errors; therefore, a two‐sided *p*‐value < 0.05 was considered statistically significant.

The global distribution map of CKM syndrome‐related ASDR and ASMR due to dietary risks in GBD was created using the ggmap (4.0.1) package, while line plots were generated using ggplot2 (3.5.1). Restricted cubic spline (RCS) regression analysis was performed using the plotRCS package (0.1.5), and the corresponding RCS plots were generated. The nomogram was constructed using the rmda package (1.6), with calibration and decision curve analysis (DCA) plots created using this package as well. Forest plots were generated using the forestploter package (1.1.2), and heatmaps were created using the heatmaps package (1.0.12).

## 3 Results

### 3.1 Study Population

GBD data were used to characterize the epidemiological burden of CKM syndrome at the population level. As the GBD study provides aggregated, publicly available estimates derived from integrated modeling rather than individual‐level participant data, no individual inclusion or exclusion criteria were applicable.

For individual‐level analyses, we used data from NHANES, which initially included 97,657 participants. Participants were sequentially excluded if they had missing data on BMI or waist circumference (*n* = 10,533), estimated eGFR or ACR (*n* = 7958), systolic blood pressure (SBP) (*n* = 5854), glycated hemoglobin (HbA1c) (*n* = 638), or total cholesterol (TC) or high‐density lipoprotein cholesterol (HDL‐C) (*n* = 1057). In addition, 23,089 participants with missing dietary intake information were excluded. After these exclusions, 48,528 participants were included in the baseline analysis to examine the associations between dietary nutrient intake and CKM syndrome prevalence.

For analyses of CKM syndrome‐related mortality, participants without available mortality follow‐up data were further excluded (*n* = 18,321), leaving 30,207 participants for the prospective mortality analysis. A detailed flowchart of participant selection is provided in Figure S2.

### 3.2 Global Disease Burden of CKM Syndrome and the Role of Diet

**FIGURE 2:**
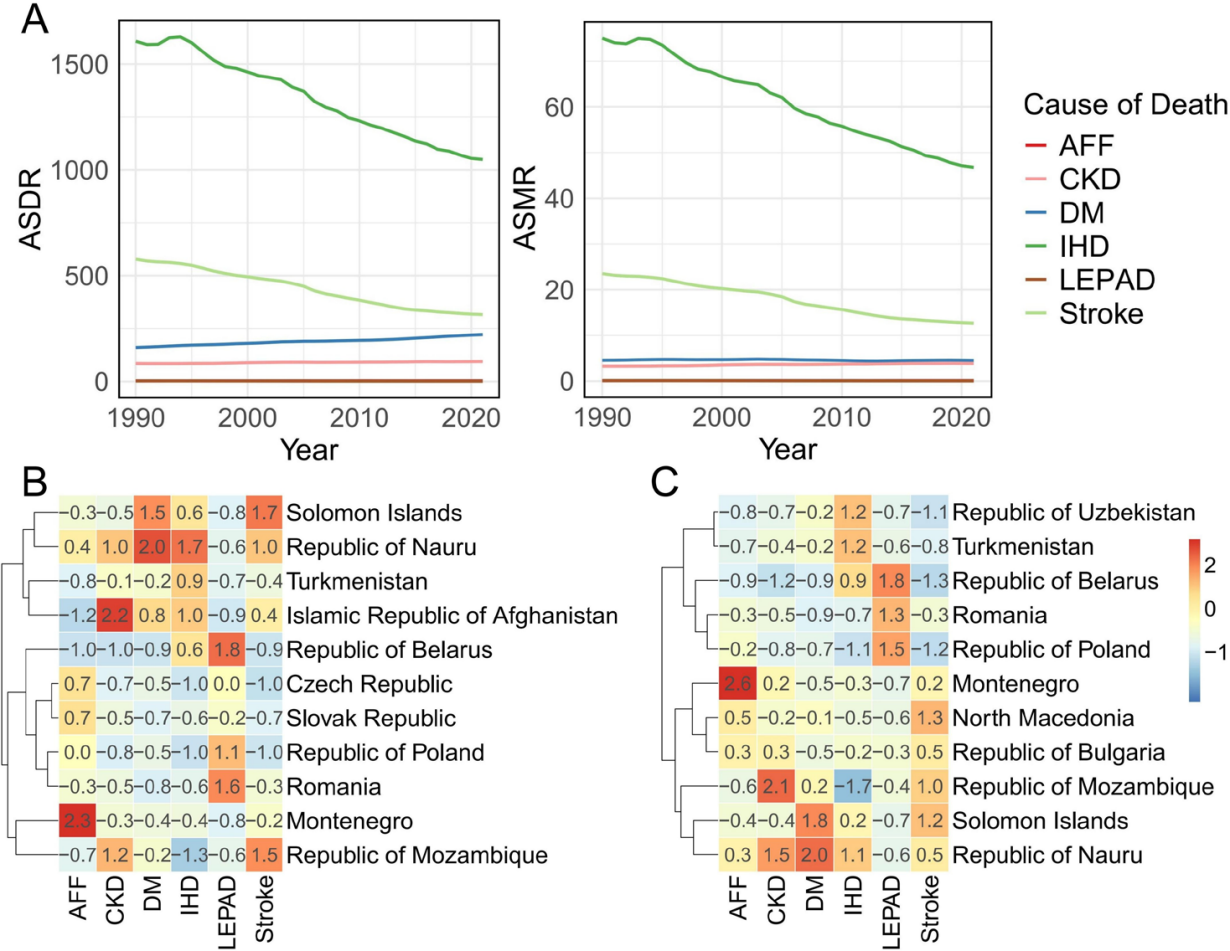
Changes in the global disease burden of CKM syndrome‐related mortality attributable to dietary risks. (A) The line plot shows the changes in ASDR and ASMR for six CKM syndrome‐related diseases attributable to dietary risks from 1990 to 2021. (B, C) Heatmaps display the ASDR (left) and ASMR (right) for the top three regions with the highest burden for each of the six CKM syndrome‐related diseases globally. Abbreviations: AFF, atrial fibrillation and flutter; CKD, chronic kidney disease; DM, diabetes mellitus; IHD, ischemic heart disease; LEPAD, lower extremity peripheral arterial disease.

From 1990 to 2021, dietary risks remained the prominent contributor to the ASMR and ASDR for IHD and stroke within CKM syndrome, with both showing a downward trend. However, ASDR and ASMR for DM and CKD showed slight upward trends. Additionally, stroke‐related ASDR and ASMR showed minimal change over the past three decades (Figure 2A). Notably, the distribution of CKM syndrome‐related ASDR and ASMR varied significantly across regions (Figures S3 and S4). Montenegro had the highest global ASDR (17.8/100,000) and ASMR (1.1/100,000) for dietary risks, while the Islamic Republic of Afghanistan had the highest CKD ASDR (267/100,000) and the Republic of Mozambique the highest CKD ASMR (8.9/100,000). For DM and IHD, the Republic of Nauru exhibited the highest ASDR (1148/100,000 for DM and 4749/100,000 for IHD) and ASMR (33.4/100,000 for DM and 0.02/100,000 for IHD). The Republic of Belarus had the highest LEPAD‐related ASDR (6.6/100,000) and ASMR (0.3/100,000). In terms of stroke, the Solomon Islands showed the highest ASDR (1255/100,000), while North Macedonia had the highest ASMR (50.9/100,000) (Figure 2B,C).

### 3.3 Baseline Characteristics and Nutrient Intake Patterns of CKM Syndrome Patients

We first analyzed the baseline characteristics and dietary patterns of 48,528 participants. The weighted average age was 44.8 years, with a significant increase in age across advancing CKM syndrome stages. While males comprised 47.4% of all participants, their proportion significantly rose to 56.2% in advanced stages. Most participants were Non‐Hispanic White (42.27%) and Non‐Hispanic Black (23.80%), with Non‐Hispanic Whites appearing more prone to progressing to advanced stages. Lifestyle factors, including FIPR, smoking, alcohol consumption, and physical activity, exhibited significant differences across CKM syndrome stages. Overall, higher FIPR, smoking, alcohol use, and lack of physical activity were notably more prevalent in advanced stages.

Among the 26 nutrients, significant differences in the intake of all macronutrients, micronutrients, and minerals were observed across the five CKM syndrome stages, as well as between non‐advanced and advanced stages. Notably, in advanced stages, the intake of other nutrients, including caffeine, was significantly higher, while the intake of PFA 20:4 was notably lower (Table 1).

**TABLE 1:** Baseline characteristics of NHANES 2001–2020 participants by CKM syndrome staging.

Characteristics	Stage 0	Stage 1	Stage 2	Stage 3	Stage 4	Nonadvanced stage	Advanced stages
Participants	2814	18,545	19,803	2542	4824	41,162	7366
Age^a^, ^b^	33.4 (0.3)	38.0 (0.2)	47.0 (0.2)	63.8 (0.5)	65.0 (0.2)	41.8 (0.1)	64.4 (0.2)
Male (%)	1171 (41.61%)	8927 (48.1%)	8785 (44.4%)	1427 (56.1%)	2715 (56.3%)	18,883 (45.9%)	4142 (56.2%)
Race							
Mexican American^a^, ^b^	433 (15.4%)	4016 (21.7%)	3588 (18.1%)	400 (15.7%)	471 (9.8%)	8037 (19.5%)	871 (11.8%)
Other Hispanic^a^, ^b^	204 (7.2%)	1648 (8.9%)	1638 (8.3%)	199 (7.8%)	286 (5.9%)	3490 (8.5%)	485 (6.6%)
Non‐Hispanic White^a^, ^b^	1214 (43.1%)	6811 (36.7%)	8251 (41.7%)	1132 (44.5%)	2744 (56.9%)	16,276 (39.5%)	3876 (52.6%)
Non‐Hispanic Black^a^	614 (21.8%)	4527 (24.4%)	4676 (23.6%)	657 (25.8%)	1074 (22.3%)	9817 (23.8%)	1731 (23.5%)
Others^a^, ^b^	349 (12.4%)	1543 (8.3%)	1650 (8.3%)	154 (6.1%)	249 (5.2%)	3542 (8.6%)	403 (5.5%)
FIPO %^a^, ^b^	3.16 (0.04)	3.01 (0.02)	2.98 (0.02)	2.80 (0.04)	2.62 (0.03)	3.01 (0.01)	2.68 (0.02)
Smoker							
Yes^a^, ^b^	639 (22.71%)	5359 (28.90%)	7237 (36.54%)	1506 (59.24%)	2956 (61.28%)	13,235 (32.15%)	4462 (60.58%)
No^a^, ^b^	1292 (45.91%)	8314 (44.83%)	9096 (45.93%)	779 (30.65%)	1864 (38.64%)	18,702 (45.48%)	2643 (35.99%)
Drinking alcohol							
Yes^a^, ^b^	1315 (46.73%)	9015 (48.61%)	10,162 (51.32%)	1436 (56.49%)	2868 (59.45%)	20,492 (49.78%)	4304 (58.43%)
No^a^, ^b^	553 (19.65%)	3996 (21.55%)	5526 (27.90%)	790 (31.08%)	1748 (36.24%)	10,075 (24.48%)	2538 (34.46%)
Physical activity							
Active^a^, ^b^	976 (34.68%)	5318 (28.68%)	4512 (22.78%)	490 (19.28%)	806 (16.71%)	10,806 (26.25%)	1296 (17.59%)
Inactive^a^, ^b^	1479 (52.56%)	9869 (53.22%)	10,945 (55.27%)	1572 (61.84%)	3044 (63.1%)	22,293 (54.16%)	4616 (62.67%)
Macronutrients (g)							
Protein^a^, ^b^	81.4 (0.9)	83.6 (0.4)	80.8 (0.3)	74.1 (0.8)	73.1 (0.6)	82.1 (0.2)	73.4 (0.5)
Carbohydrates^a^, ^b^	266.7 (2.7)	261.7 (1.1)	250.2 (1.1)	225.0 (2.3)	227.8 (1.9)	256.7 (0.7)	226.9 (1.5)
Total sugar^a^, ^b^	120.8 (1.6)	119.1 (0.7)	112.6 (0.7)	97.0 (1.5)	102.8 (1.2)	116.2 (0.5)	100.9 (0.9)
Dietary fiber^a^, ^b^	17.0 (0.2)	16.4 (0.1)	16.2 (0.1)	16.3 (0.2)	15.4 (0.2)	16.4 (0.1)	15.7 (0.1)
Total fat^a^, ^b^	81.0 (0.9)	83.4 (0.4)	80.2 (0.4)	74.2 (0.9)	73.5 (0.7)	81.7 (0.3)	73.7 (0.6)
Cholesterol^a^, ^b^	268.7 (5.4)	290.3 (2.2)	286.2 (1.8)	276.6 (4.7)	271.3 (3.5)	286.7 (1.5)	273.0 (2.8)
Saturated fatty acids^a^, ^b^	26.4 (0.3)	27.4 (0.1)	26.4 (0.1)	24.4 (0.3)	23.8 (0.3)	26.9 (0.1)	24.0 (0.2)
Micronutrients							
Total Vitamin A (μg)^a^	645.6 (12.5)	620.7 (6.7)	631.1 (5.2)	658.0 (12.7)	620.3 (10.9)	627.4 (4.0)	632.4 (8.5)
α‐Carotene (μg)^a^	443.7 (22.7)	396.8 (16.1)	394.4 (8.6)	424.6 (24.9)	357.8 (12.2)	399.2 (8.6)	379.2 (11.5)
β‐Carotene (μg)^a^	2316.8 (82.3)	2052.3 (54.5)	2103.7 (29.6)	2284.1 (89.1)	2006.0 (49.2)	2096.1 (29.2)	2095.4 (44.1)
Niacin (mg)^a^, ^b^	25.5 (0.3)	25.7 (0.1)	24.6 (0.1)	22.6 (0.3)	22.4 (0.2)	25.2 (0.1)	22.5 (0.2)
Vitamin B6 (mg)^a^, ^b^	2.1 (0.0)	2.1 (0.0)	2.0 (0.0)	1.9 (0.0)	1.9 (0.0)	2.0 (0.0)	1.9 (0.0)
Total folate (μg)^a^, ^b^	422.8 (5.6)	407.0 (2.2)	398.2 (2.1)	373.0 (4.4)	367.5 (4.0)	404.1 (1.5)	369.2 (3.0)
Vitamin B12 (mg)^a^	1.0 (0.1)	0.9 (0.0)	0.9 (0.0)	0.8 (0.0)	0.9 (0.1)	0.9 (0.0)	0.8 (0.0)
Vitamin C (mg)^a^, ^b^	87.1 (1.9)	82.2 (0.8)	82.1 (0.7)	80.0 (1.7)	80.0 (1.5)	82.5 (0.5)	80.0 (1.1)
Vitamin D (μg)^a^	2.9 (0.1)	3.2 (0.0)	3.1 (0.0)	3.5 (0.1)	3.1 (0.1)	3.2 (0.0)	3.2 (0.1)
Vitamin K (μg)^a^	108.7 (3.1)	107.8 (3.8)	102.6 (1.2)	104.3 (2.7)	98.6 (2.1)	105.4 (1.9)	100.4 (1.7)
Vitamin E (mg)^a^, ^b^	9.1 (0.2)	8.7 (0.1)	8.4 (0.1)	8.1 (0.2)	7.9 (0.1)	8.6 (0.1)	8.0 (0.1)
Total choline (mg)^a^	223.5 (4.8)	260.3 (1.9)	252.6 (1.8)	267.8 (4.4)	245.1 (3.5)	253.9 (1.2)	252.4 (2.8)
Minerals (mg)							
Calcium^a^, ^b^	968.3 (13.1)	966.6 (5.1)	934.9 (5.1)	865.7 (12.0)	835.3 (8.8)	951.9 (3.4)	845.1 (7.1)
Phosphorus^a^, ^b^	1367.1 (14.2)	1386.4 (5.8)	1341.4 (5.7)	1249.6 (12.9)	1218.9 (10.4)	1364.0 (3.9)	1228.8 (8.2)
Sodium^a^, ^b^	3472.4 (36.1)	3523.0 (14.8)	3412.4 (14.7)	3150.9 (35.1)	3126.3 (28.7)	3467.5 (10.0)	3134.2 (22.5)
Potassium^a^, ^b^	2663.5 (27.6)	2640.7 (11.6)	2625.0 (10.5)	2583.0 (26.1)	2549.2 (20.4)	2635.0 (7.5)	2560.1 (16.2)
Other nutrients							
Caffeine (mg)^b^	146.4 (5.4)	155.5 (2.0)	157.5 (1.9)	166.7 (5.3)	178.2 (4.2)	155.8 (1.3)	174.5 (3.3)
PFA 20:4 (g)^b^	1.3 (0.0)	1.5 (0.0)	1.5 (0.0)	1.4 (0.0)	1.3 (0.0)	0.14 (0.0)	0.13 (0.0)
PFA 20:5 (g)	0.03 (0.0)	0.04 (0.0)	0.03 (0.0)	0.03 (0.0)	0.03 (0.0)	0.03 (0.0)	0.03 (0.0)

*Note:* Categorical variables are presented as frequencies (percentages), while continuous variables are presented as means (SE). Categorical variables were analyzed using survey‐weighted Pearson *χ* ^2^ test, and continuous variables were analyzed using survey‐weighted linear regression.

Abbreviation: FIPO, family income to poverty ratio.

^a^ Indicates significant differences across all five CKM stages.

^b^ Indicates significant differences between advanced and non‐advanced CKM stages (*p* < 0.05).

### 3.4 Relationship Between Nutrient Intake and CKM Syndrome Staging

After adjusting for confounding factors, including age, gender, race, FIPR, smoking, alcohol consumption, and physical activity, we found that among macronutrients, the intake of saturated fatty acids significantly reduced the risk of CKM syndrome stage 2 (RR: 0.82, 95% CI: 0.69–0.97) and stage 4 (RR: 0.77, 95% CI: 0.62–0.95). Among micronutrients, vitamin D intake significantly reduced the risk of stage 2 (RR: 0.90, 95% CI: 0.84–0.97), while vitamin E intake significantly lowered the risk of stages 1 (RR: 0.90, 95% CI: 0.85–0.95), 2 (RR: 0.91, 95% CI: 0.86–0.96), and 4 (RR: 0.89, 95% CI: 0.82–0.96). Among minerals, phosphorus intake effectively reduced the risk of stage 4 (RR: 0.69, 95% CI: 0.54–0.89). Notably, potassium intake was associated with reduced risk across stages 1–4. Other nutrients, including caffeine and PFA, showed no significant association with CKM syndrome staging (Table 2).

**TABLE 2:** Relative risk of 26 nutrients for CKM syndrome staging.

Characteristics	RR(95 CI%) of CKM syndrome^a^, Stage 0 as the Reference			
	Stage 1	Stage 2	Stage 3	Stage 4
Macronutrients (g)				
Protein	1.04 (0.87–1.24)	1.10 (0.92–1.32)	1.03 (0.79–1.34)	1.07 (0.85–1.34)
Carbohydrates	0.90 (0.74–1.09)	1.03 (0.84–1.25)	0.99 (0.74–1.32)	0.96 (0.75–1.23)
Total sugars	1.10 (0.95–1.27)	0.97 (0.84–1.13)	0.84 (0.68–1.06)	1.04 (0.87–1.25)
Dietary fiber	1.04 (0.94–1.14)	0.94 (0.85–1.04)	1.03 (0.90–1.18)	0.96 (0.85–1.09)
Total fat	1.21 (1.01–1.46)	1.17 (0.97–1.42)	1.19 (0.91–1.56)	1.33 (1.05–1.67)
Saturated fatty acids	0.87 (0.73–1.03)	0.82 (0.69–0.97)	0.79 (0.62–1.01)	0.77 (0.62–0.95)
Micronutrients				
Total Vitamin A (μg)	0.93 (0.85–1.03)	0.97 (0.89–1.07)	0.92 (0.82–1.04)	0.99 (0.90–1.10)
α‐Carotene (μg)	1.05 (0.98–1.13)	1.03 (0.96–1.11)	1.05 (0.95–1.16)	0.97 (0.88–1.06)
β‐Carotene (μg)	0.95 (0.86–1.06)	0.96 (0.87–1.07)	0.99 (0.86–1.13)	0.94 (0.83–1.06)
Niacin (mg)	1.05 (0.92–1.21)	1.04 (0.90–1.20)	1.04 (0.84–1.28)	0.96 (0.80–1.14)
Vitamin B6 (mg)	1.05 (0.93–1.18)	0.97 (0.86–1.10)	1.04 (0.87–1.25)	1.04 (0.89–1.21)
Total folate (μg)	1.05 (0.96–1.14)	1.01 (0.93–1.11)	0.93 (0.81–1.08)	0.97 (0.86–1.09)
Vitamin B12 (mg)	0.96 (0.89–1.04)	1.01 (0.93–1.09)	0.93 (0.82–1.06)	0.99 (0.90–1.10)
Vitamin C (mg)	0.99 (0.93–1.07)	1.01 (0.94–1.08)	0.96 (0.86–1.07)	1.04 (0.95–1.14)
Vitamin D (μg)	0.95 (0.88–1.01)	0.90 (0.84–0.97)	0.91 (0.83–1.00)	0.95 (0.87–1.04)
Vitamin K (μg)	1.05 (0.99–1.10)	0.98 (0.92–1.05)	0.99 (0.90–1.09)	0.96 (0.88–1.05)
Vitamin E (mg)	0.90 (0.85–0.95)	0.91 (0.86–0.96)	0.92 (0.84–1.00)	0.89 (0.82–0.96)
Total choline (mg)	1.13 (1.05–1.23)	1.13 (1.04–1.23)	1.35 (1.19–1.52)	1.09 (0.98–1.21)
Minerals (mg)				
Calcium	1.24 (1.10–1.39)	1.34 (1.18–1.51)	1.43 (1.20–1.69)	1.41 (1.21–1.63)
Phosphorus	0.89 (0.73–1.08)	0.86 (0.70–1.05)	0.87 (0.65–1.16)	0.69 (0.54–0.89)
Sodium	0.97 (0.88–1.08)	1.10 (0.99–1.23)	1.09 (0.93–1.27)	1.18 (1.03–1.35)
Potassium	0.70 (0.61–0.81)	0.72 (0.63–0.83)	0.63 (0.52–0.77)	0.67 (0.56–0.80)
Other nutrients				
Caffeine (mg)	1.04 (0.97–1.11)	0.99 (0.93–1.06)	1.04 (0.95–1.13)	1.05 (0.97–1.13)
PFA 20:4 (g)	1.12 (1.02–1.23)	1.14 (1.04–1.25)	1.15 (1.02–1.30)	1.21 (1.08–1.35)
PFA 20:5 (g)	0.99 (0.94–1.06)	1.00 (0.95–1.06)	1.03 (0.95–1.12)	1.00 (0.93–1.08)

^a^ After Z‐standardizing the 26 nutrients, multinomial logistic regression was performed to analyze the association between nutrient intake and CKM Syndrome stages, adjusting for potential confounders including age, sex, race, income, smoking, alcohol consumption, and physical activity. The relative RR and 95% CIs for each CKM Syndrome stage were calculated.

### 3.5 Association Between Sodium Intake and Advanced Stages of CKM Syndrome

**FIGURE 3:**
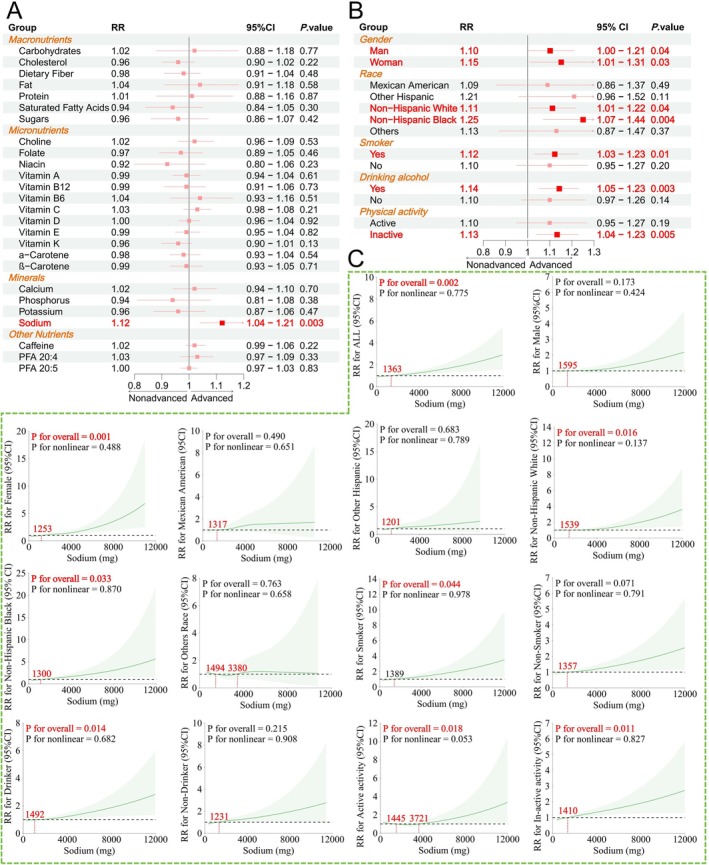
Regression and subgroup analysis of 26 nutrients and CKM syndrome advanced stages. (A) Forest plot showing the weighted Poisson regression analysis of 26 nutrients and their association with CKM syndrome advanced stages. (B) Forest plot depicting the subgroup analysis of sodium intake and CKM syndrome advanced stages, stratified by gender, race, smoking, alcohol consumption, and physical activity. (C) RCS regression analysis of sodium intake and CKM syndrome advanced stages, along with subgroup analyses for each group. The plot includes the *P*‐value for the overall effect and the *P*‐value for the nonlinear effect.

Using weighted Poisson regression, we found that among the 26 nutrients, only sodium intake significantly influenced the progression to advanced stages of CKM syndrome after adjusting for all confounders (RR: 1.12, 95% CI: 1.04–1.21, *p* = 0.003; Figure 3A). Subgroup analysis further revealed that the association between sodium intake and advanced CKM syndrome stages remained significant in both males (RR: 1.10, 95% CI: 1.00–1.21, *p* = 0.04) and females (RR: 1.15, 95% CI: 1.01–1.31, *p* = 0.03). However, with respect to race, this association was significant only in Non‐Hispanic White (RR: 1.11, 95% CI: 1.01–1.22, *p* = 0.04) and Non‐Hispanic Black populations (RR: 1.25, 95% CI: 1.07–1.44, *p* = 0.004). Interestingly, the impact of sodium intake on the progression to advanced CKM syndrome stages was also significant among smokers (RR: 1.12, 95% CI: 1.03–1.23, *p* = 0.01), alcohol consumers (RR: 1.14, 95% CI: 1.05–1.23, *p* = 0.003), and participants with low physical activity (RR: 1.10, 95% CI: 1.04–1.23, *p* = 0.005; Figure 3B).

We then conducted an RCS regression analysis to further examine the impact of sodium intake (Figure 3C). In the overall population, sodium intake was significantly associated with advanced stages of CKM syndrome (*p* = 0.002), with a RR exceeding 1 when sodium intake exceeded 1363 mg, suggesting a harmful effect. Furthermore, significant associations were observed in females (*p* = 0.001), Non‐Hispanic White (*p* = 0.016) and Black populations (*p* = 0.033), as well as among smokers (*p* = 0.044), alcohol consumers (*p* = 0.014), and individuals with low physical activity (*p* = 0.011). Interestingly, RCS regression also revealed a significant association between sodium intake and advanced CKM syndrome stages in physically active individuals (*p* = 0.018). A U‐shaped relationship was observed, where sodium intake between 1445 and 3721 mg was associated with an RR less than 1, indicating a potentially protective effect, although the *P*‐value was not statistically significant.

### 3.6 Association Between Nutrient Intake and CKM Syndrome Mortality Risk, and Model Construction

**FIGURE 4:**
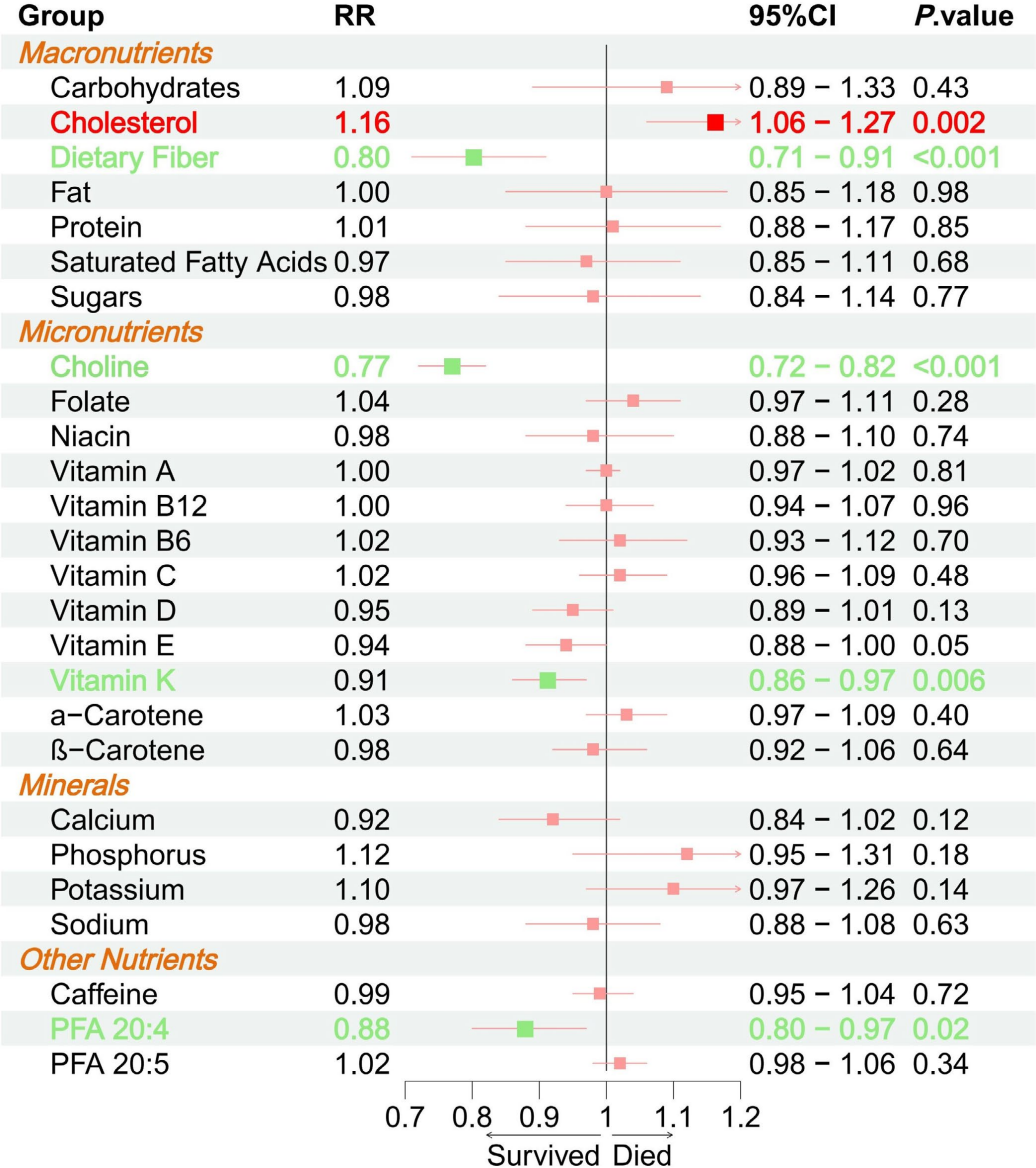
Forest plot of weighted Poisson regression analysis of 26 nutrients and all‐cause mortality risk in CKM syndrome.

Analysis of mortality outcomes in 30,207 participants revealed a significant increase in all‐cause mortality with advancing CKM syndrome stages, from 5.71% in stage 0 to 51.35% in stage 4 (*p* < 0.001). The proportion of deaths attributable to CKM syndrome also increased significantly, with 21.78% of deaths in advanced stages due to CKM syndrome, compared to only 3.48% in non‐advanced stages (*p* < 0.001; Table S1). Multivariable weighted regression analysis showed that cholesterol intake was significantly associated with increased all‐cause mortality risk (RR: 1.16, 95% CI: 1.06–1.27, *p* = 0.002), while dietary fiber (RR: 0.80, 95% CI: 0.71–0.91, *p* < 0.001), choline (RR: 0.77, 95% CI: 0.72–0.82, *p* < 0.001), vitamin K (RR: 0.91, 95% CI: 0.86–0.97, *p* = 0.006), and PFA 20:4 (RR: 0.88, 95% CI: 0.80–0.97, *p* = 0.02) intake were significantly associated with reduced all‐cause mortality risk in CKM syndrome (Figure 4). Additionally, factors such as age, gender, race, smoking, alcohol consumption, and FIPR were also found to be associated with CKM syndrome mortality risk (Table S2). After adjusting for confounders, no significant association was found between nutrient intake and CKM syndrome‐related mortality risk (Table S3).

**FIGURE 5:**
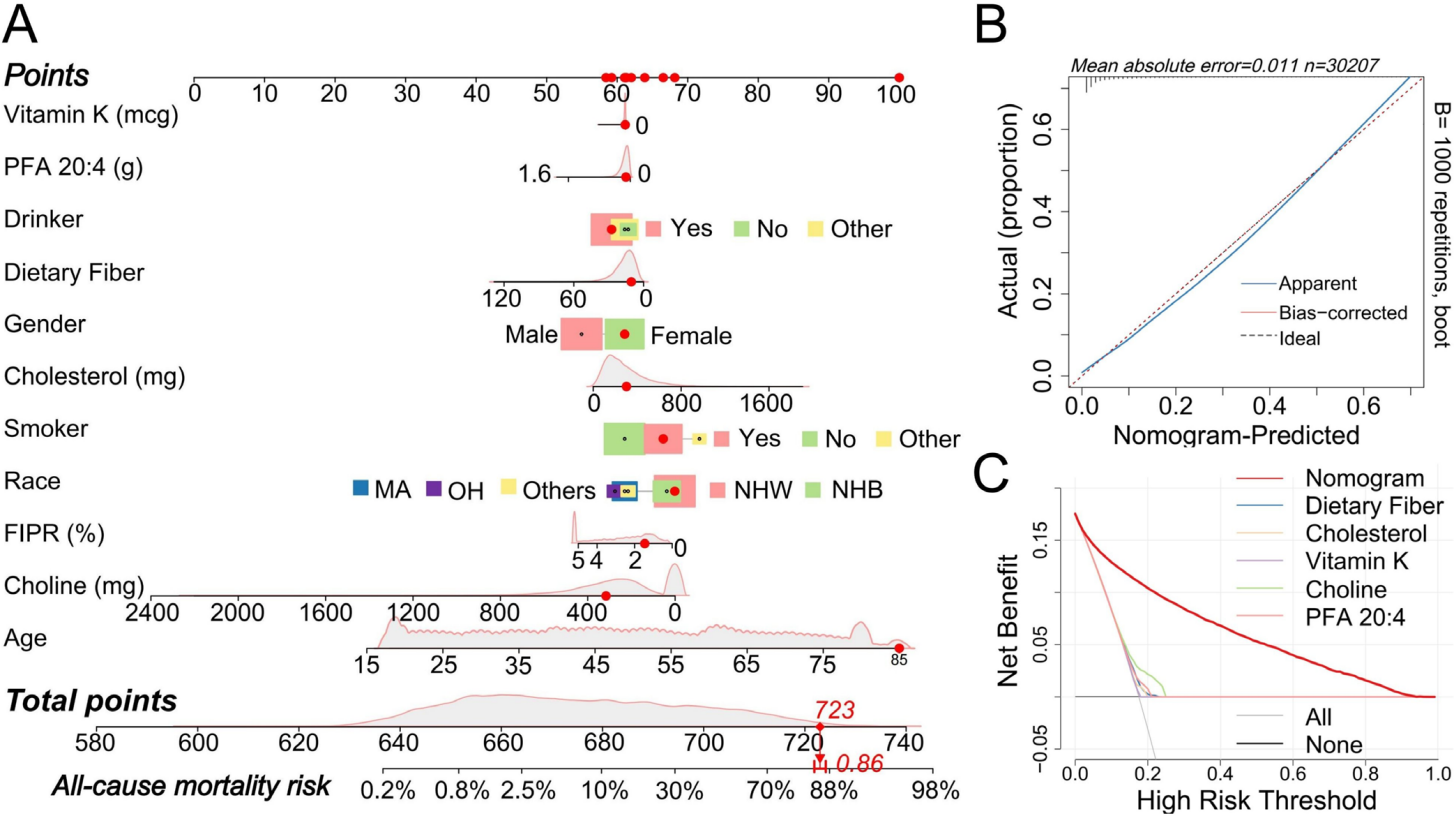
Construction and validation of the nutrient‐based CKM syndrome mortality risk prediction model. (A) Nomogram for predicting CKM syndrome mortality risk based on five nutrients, along with age, sex, race, smoking, alcohol consumption, and FIPR. Randomly selected, the 10,000th participant's risk score is shown in the plot, with the final predicted all‐cause mortality risk for this participant being 0.86. (B) Calibration curve for the nomogram. The “Apparent” curve represents the observed performance of the model, the “Bias‐corrected” curve reflects the model's performance after correcting for bias, and the “Ideal” curve represents perfect prediction. (C) DCA comparing the performance of the model using five individual nutrients versus the nomogram. The DCA shows the net benefit of using the model at different thresholds of CKM syndrome mortality risk, allowing comparison between individual nutrient models and the integrated nomogram approach.

Subsequently, we incorporated the five nutrients significantly associated with all‐cause CKM syndrome mortality, along with significant confounders, to construct a nomogram for predicting all‐cause CKM syndrome mortality risk (Figure 5A). The calibration curve of the nomogram showed a good fit (Figure 5B), and the DCA confirmed that the nomogram model provides a net benefit across a wide range of high‐risk thresholds (Figure 5C).

## 4 Discussion

CKM syndrome remains a significant global health burden, with a long treatment and management cycle, leading to high healthcare costs and substantial pressure on individuals and society. It is associated with millions of new cases and over 20 million deaths annually, underscoring the urgent need for effective prevention strategies (Xie et al. 2025). Early intervention and prevention are key to addressing CKM syndrome. As a core modifiable risk factor in the development and progression of CKM syndrome, diet warrants further exploration. Our comprehensive study identifies associations between low potassium and high sodium intake with advanced CKM syndrome, while increased cholesterol intake is linked to higher all‐cause mortality. Conversely, higher intakes of dietary fiber, choline, vitamin K, and PFA 20:4 were found to be protective.

The GBD analysis conducted in this study reveals a significant decline in ASDR and ASMR for IHD and stroke caused by dietary risks over the past 30 years, which can be attributed to advances in antihypertensive treatment and statin use, as well as improvements in cardiovascular disease management and patient care (Roth et al. 2020). However, deaths from CKD, DM, lower extremity peripheral arterial disease, and AFF due to dietary risks have not yet significantly improved, likely due to increasing patient numbers, aging populations, and inadequate early screening (Ying et al. 2024; Zhang et al. 2025; Wang, Yu, et al. 2024). Furthermore, the global variation in CKM syndrome‐related mortality from dietary risks is striking, with higher rates observed in regions like Europe, particularly in the Republic of Belarus and the Republic of Poland, which are closely associated with dietary habits, including high intake of animal fats, sugars, and sodium (Liang et al. 2023; Xie et al. 2024). These findings highlight the necessity of dietary interventions, particularly in regions with a high disease burden, to address the global impact of CKM syndrome.

Risk factors for CKM syndrome, including age, gender, and race, have been extensively studied and well established, and our cohort further supports these findings (Xie et al. 2025; Zhu et al. 2024). However, the role of nutrient intake in CKM syndrome remains unclear. Our comparison reveals significant differences in nutrient intake across CKM syndrome stages, ranging from macronutrients like sugars, fats, and proteins to micronutrients such as vitamins and minerals. Interestingly, reduced potassium intake was associated with an increased risk of advancing from stage 0 to stages 1–4 of CKM syndrome. Potassium intake is closely linked to cardiovascular and kidney health (Stone et al. 2016). It helps lower blood pressure by promoting sodium excretion, inhibiting the renin‐angiotensin‐aldosterone system (RAAS), and relaxing vascular smooth muscle (Weaver 2013). Potassium regulates endothelial oxidative stress, inflammation (Ekun et al. 2020), and insulin sensitivity in skeletal muscles (Rodríguez‐Rivera and Barrera‐Oviedo 2024) Dietary potassium supplementation provides a low‐cost, effective measure for preventing and managing CKM syndrome.

After adjusting for age, gender, and other factors, only sodium intake was significantly associated with advanced stages of CKM syndrome. Sodium intake and management have long been critical topics in CKM syndrome‐related diseases. A high‐sodium diet activates the RAAS system and sympathetic nervous system, which leads to increased systemic blood pressure and glomerular filtration rate, and significantly worsens ventricular hypertrophy (Nishimoto et al. 2024). High sodium intake is closely linked to insulin resistance (Baudrand et al. 2014). However, a low‐sodium diet, such as the DASH diet, has been shown to effectively reduce the risk of CKM syndrome‐related diseases and slow their progression (Jeong et al. 2023; Akubo et al. 2025), making it a core measure in CKM syndrome dietary management. Interestingly, subgroup analysis revealed that the association between sodium intake and advanced CKM syndrome stages varied significantly across populations. This association remained significant in Non‐Hispanic White and Non‐Hispanic Black populations but was not observed in Mexican American or other ethnic groups, possibly due to differences in sodium sensitivity and genetic factors (Rudnicki and Mayer 2009; Mahajan et al. 2016). Furthermore, the association between sodium intake and CKM syndrome progression was also present in smokers, alcohol consumers, and individuals with low physical activity. Smoking and alcohol consumption can lead to increased sodium retention and sensitivity (Liu et al. 2025; Krittanawong et al. 2022). Lack of physical activity is also closely associated with these factors (Wilund et al. 2021), and their cumulative effect, when combined with high sodium intake, accelerates CKM syndrome progression.

Additionally, we found a U‐shaped relationship between sodium intake and the risk of advanced CKM syndrome in physically active individuals. Specifically, sodium intake between 1445 and 3721 mg was associated with a protective effect. Adequate sodium intake is essential for maintaining electrolyte balance and cardiovascular function. A moderate increase in sodium intake may help sustain cardiovascular and kidney health in physically active individuals, defined as those engaging in ≥ 150 min of moderate‐intensity or ≥ 75 min of vigorous‐intensity physical activity per week. This level of physical activity enhances the body's ability to adapt to and regulate sodium, thereby reducing CKM syndrome risk (Wang et al. 2025; Baker‐Smith et al. 2025). RCS regression further revealed that the critical threshold for sodium intake in relation to CKM syndrome progression risk varies across populations. For instance, in males, it is approximately 1595 mg, while in females, it is around 1253 mg. This suggests that sodium intake management may not be absolute but should be tailored to individual characteristics for better control.

Furthermore, this study shows increased cholesterol intake significantly elevated the all‐cause mortality risk in CKM syndrome individuals. High cholesterol intake also increases low‐density lipoprotein (LDL) cholesterol, which has been established as an independent risk factor for CVD (Wang et al. 2023). Elevated cholesterol can also lead to endothelial damage and atherosclerosis, increasing the risk of heart disease, stroke, and CKD (Tabas 2002). In addition, cholesterol deposition in the kidneys severely impairs renal tubular function, promotes kidney fibrosis, and ultimately contributes to patient mortality. In contrast, dietary fiber intake was significantly associated with reduced all‐cause mortality risk in CKM syndrome (Mitrofanova et al. 2023). Dietary fiber plays a critical role in the body by effectively regulating blood lipids, lowering cholesterol levels, delaying glucose release, enhancing insulin sensitivity, and potentially exerting anti‐inflammatory effects through modulation of the gut microbiota (Fuller et al. 2016; Makki et al. 2018). The intake of dietary fiber can potentially reduce CKM syndrome‐related mortality risk. Choline and vitamin K intake were also significantly associated with reduced CKM syndrome‐related mortality risk. Choline, a key component in phospholipid synthesis, is vital for the health of the heart, brain, and kidneys. It also effectively lowers homocysteine levels, which may delay CKM syndrome progression and reduce mortality (Kansakar et al. 2023; Wood and Allison 1982). Vitamin K plays a crucial role in regulating calcium metabolism and preventing atherosclerosis (Kaesler et al. 2021). Additionally, PFA 20:4, a polyunsaturated fatty acid, helps modulate inflammation, vascular function, and lipid metabolism, providing protective effects against CKM syndrome (Zhou et al. 2021; Burns et al. 2018). Adjusting nutrient intake through diet may, therefore, help reduce mortality risk in CKM syndrome patients.

Based on key characteristics such as age and gender, we constructed a predictive model for all‐cause CKM syndrome mortality risk using five nutrients significantly associated with CKM syndrome‐related death. The model demonstrated strong performance and accuracy, offering a practical and user‐friendly tool for assessing CKM syndrome mortality risk. In clinical practice, this model could be used to identify high‐risk individuals, enabling clinicians to prioritize interventions such as personalized dietary modifications, lifestyle recommendations, and pharmacological treatments. Furthermore, the simplicity of the model facilitates its integration into routine clinical assessments, allowing healthcare providers to easily monitor at‐risk populations. In research, the model could help identify key dietary factors contributing to CKM syndrome‐related mortality, guiding future studies on prevention and intervention strategies. Overall, this tool has the potential to support both individualized patient care and broader public health strategies aimed at reducing CKM syndrome‐related mortality.

Indeed, this study has several limitations. First, the interview data may be subject to reporting bias, potentially leading to inaccuracies in the results. Second, as a cross‐sectional study, it cannot establish causal relationships, and there is a lack of external cohort validation. Future research will need to be conducted in other cohorts to further validate these findings and explore causality. Limitations aside, this study is the first comprehensive investigation to elucidate the relationship between dietary nutrient intake and the incidence and mortality of CKM syndrome, based on a large‐scale population cohort. By utilizing both GBD and NHANES data, this research provides novel insights into the potential role of diet in the progression and fatality of CKM syndrome. The large sample size and the inclusion of both prevalence and mortality data enhance the robustness and generalizability of the findings.

## Author Contributions

**Sayna Norouzi:** resources, supervision, writing – review and editing. **Kaili Qin:** formal analysis, data curation, investigation, methodology. **Mingjing Gao:** validation, software. **Junnan Wu:** writing – review and editing, project administration, resources, funding acquisition, supervision. **Yimiao Ma:** validation, investigation. **Jiaying Hu:** data curation, formal analysis, methodology, investigation. **Jianbo Qing:** conceptualization, methodology, investigation, visualization, formal analysis, writing – original draft.

## Funding

This study was supported by the National Natural Science Foundation of China under grant no. 82370717, and the Zhejiang Provincial Natural Science Foundation of China under grant no. LR26H050001.

## Ethics Statement

This study utilized previously published and publicly available data, which had been ethically approved at the time of data collection. Therefore, no additional ethical approval was required for this study.

## Conflicts of Interest

The authors declare no conflicts of interest.

## Supporting information


**Figure S1:** Directed acyclic graph (DAG) for confounder selection. This DAG illustrates the assumed causal relationships between dietary nutrient intake (exposure) and CKM syndrome (outcome). Variables such as age, gender, race/ethnicity, socioeconomic status (SES), smoker, alcohol consumption, and physical activity are considered potential confounders. These confounders were identified a priori as common causes of both the exposure and the outcome and were included in the multivariable models to block backdoor paths and minimize bias in the estimation of the exposure‐outcome relationship.
**Figure S2:** Participant selection and details of the NHANES cross‐sectional study. Among 97,687 participants from 2001 to 2020, individuals missing data on BMI, eGFR, ACR, SBP, HbA1c, TC, HDL, or key nutrient intake were excluded. A total of 48,528 participants were retained for the study. Of these, 30,207 participants with complete mortality outcome data were included in the mortality risk analysis. ACR, albumin‐to‐creatinine ratio; BMI, body mass index; eGFR, estimated glomerular filtration rate; HbA1c, hemoglobin A1c; HDL, high‐density lipoprotein; SBP, systolic blood pressure; TC, total cholesterol.
**Figure S3:** Global distribution of ASDR for six CKM‐related diseases attributable to dietary risks. The world map illustrates the distribution of age‐standardized disability‐adjusted life years (ASDR) for six CKM‐related diseases. Darker colors indicate higher ASDR values. AFF, atrial fibrillation and flutter; CKD, chronic kidney disease; DM, diabetes mellitus; IHD, ischemic heart disease; LEPAD, lower extremity peripheral arterial disease.
**Figure S4:** Global distribution of ASDR for six ckm‐related diseases attributable to dietary risks. The world map illustrates the distribution of age‐standardized mortality rates (ASDR) for six CKM‐related diseases. Darker colors indicate higher ASMR values. AFF, atrial fibrillation and flutter; CKD, chronic kidney disease; DM, diabetes mellitus; IHD, ischemic heart disease; LEPAD, Lower Extremity Peripheral Arterial Disease.

**Table S1:** All‐cause mortality and CKM‐related mortality by CKM staging, with proportions and difference analysis.
**Table S2:** Relative risk (RR) of all‐cause mortality in CKM syndrome by participant demographic characteristics.
**Table S3:** Relative risk (RR) of CKM syndrome‐cause mortality in CKM syndrome by participant demographic characteristics.

## Data Availability

The data that support the findings of this study are available on request from the corresponding author. The data are not publicly available due to privacy or ethical restrictions.
