# Supplementary material for: A Comprehensive Analysis of the Impact of Nutrient Intakes on the Stages and Mortality of Cardiovascular‐Kidney‐Metabolic Syndrome
Source: Food Sci Nutr. 2026 Apr 13;14(4):e71747. doi: 10.1002/fsn3.71747 (PMC13071469; doi:10.1002/fsn3.71747)

**Supplementary information**

**Supplementary Figures**

**Figure S1. Directed Acyclic Graph (DAG) for Confounder Selection.** This DAG illustrates the assumed causal relationships between dietary nutrient intake (exposure) and CKM syndrome (outcome). Variables such as age, gender, race/ethnicity, socioeconomic status (SES), smoker, alcohol consumption, and physical activity are considered potential confounders. These confounders were identified a priori as common causes of both the exposure and the outcome and were included in the multivariable models to block backdoor paths and minimize bias in the estimation of the exposure-outcome relationship.


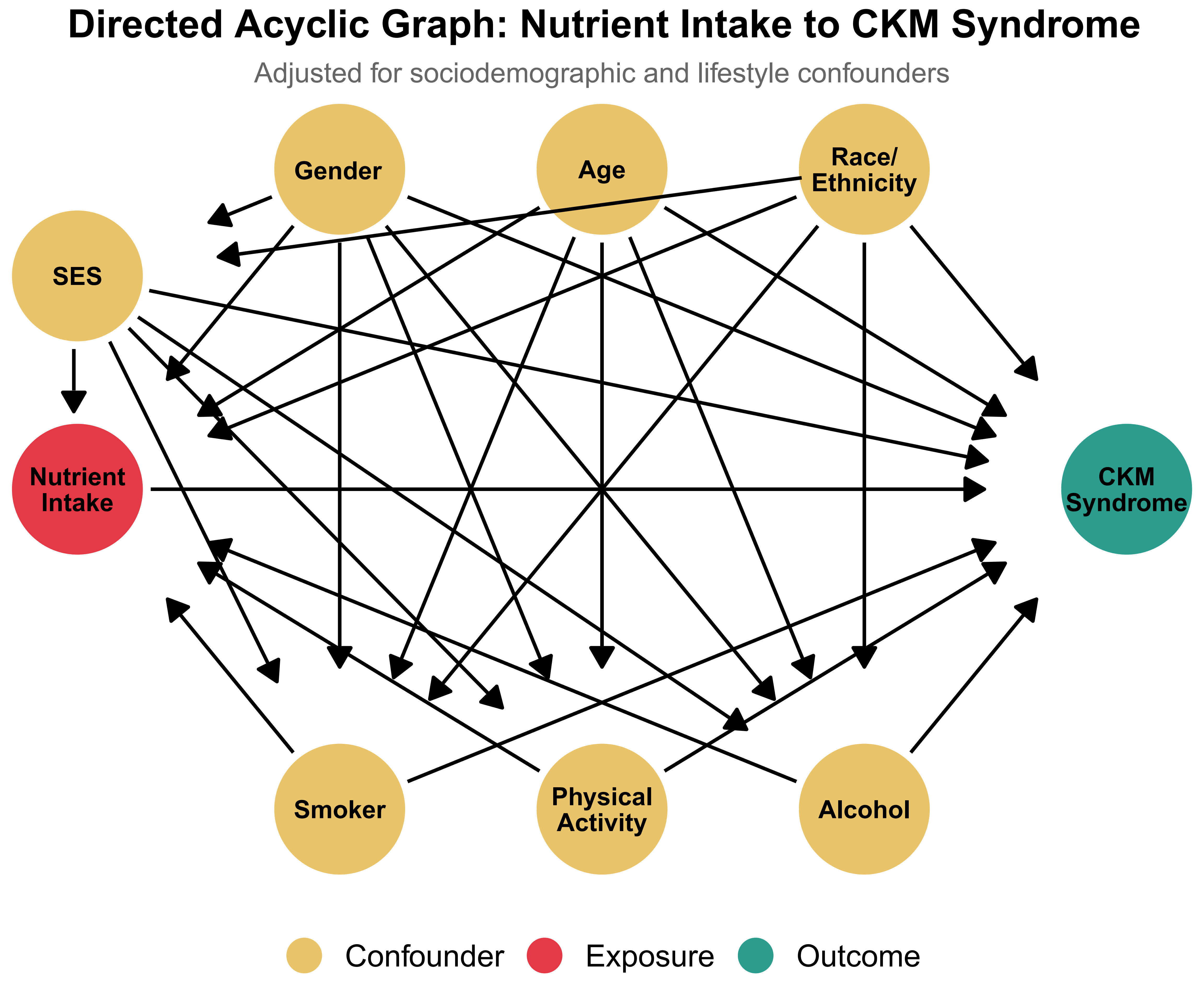


**Figure S2. Participant Selection and Details of the NHANES Cross-Sectional Study.** Among 97,687 participants from 2001-2020, individuals missing data on BMI, eGFR, ACR, SBP, HbA1c, TC, HDL, or key nutrient intake were excluded. A total of 48,528 participants were retained for the study. Of these, 30,207 participants with complete mortality outcome data were included in the mortality risk analysis. Abbreviations: BMI, Body Mass Index; eGFR, estimated Glomerular Filtration Rate; ACR, Albumin-to-Creatinine Ratio; SBP, Systolic Blood Pressure; HbA1c, Hemoglobin A1c; TC, Total Cholesterol; HDL, High-Density Lipoprotein.

**
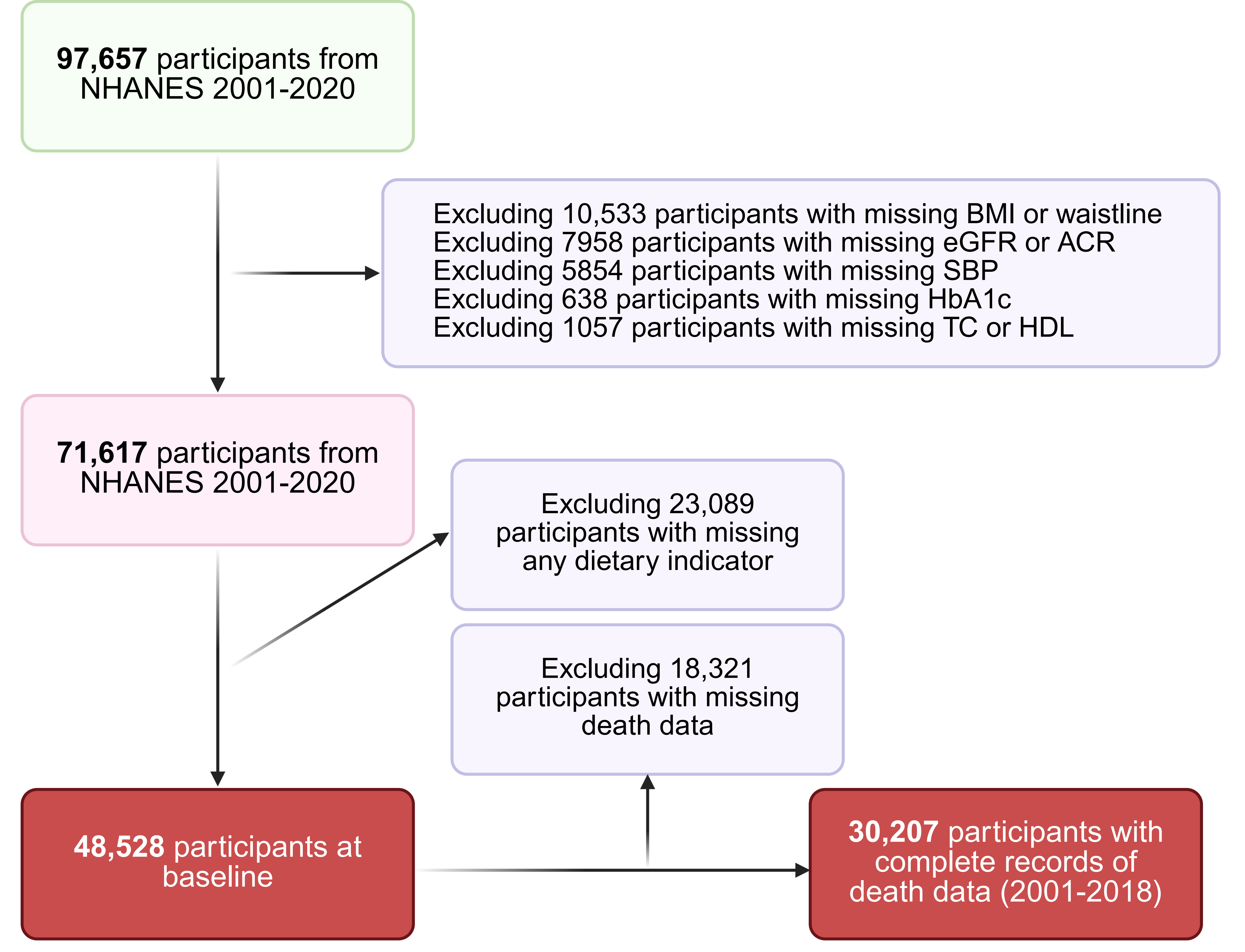
**

**Figure S3. Global Distribution of ASDR for Six CKM-Related Diseases Attributable to Dietary Risks.** The world map illustrates the distribution of age-standardized disability-adjusted life years (ASDR) for six CKM-related diseases. Darker colors indicate higher ASDR values. Abbreviations: AFF, Atrial Fibrillation and Flutter; CKD, Chronic Kidney Disease; DM, Diabetes Mellitus; IHD, Ischemic Heart Disease; LEPAD, Lower Extremity Peripheral Arterial Disease.


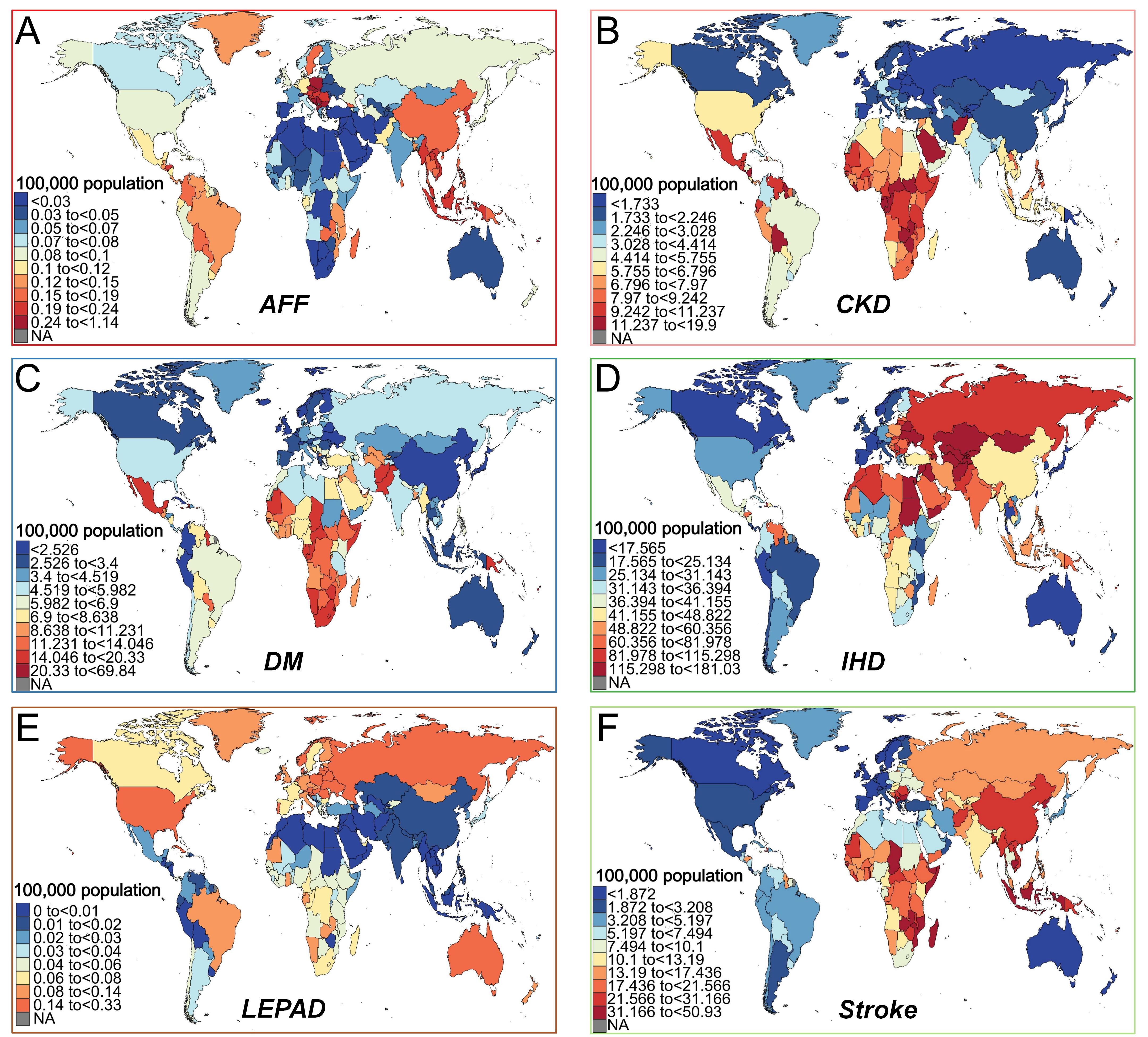


**Figure S4. Global Distribution of ASDR for Six CKM-Related Diseases Attributable to Dietary Risks.** The world map illustrates the distribution of age-standardized mortality rates (ASDR) for six CKM-related diseases. Darker colors indicate higher ASMR values. Abbreviations: AFF, Atrial Fibrillation and Flutter; CKD, Chronic Kidney Disease; DM, Diabetes Mellitus; IHD, Ischemic Heart Disease; LEPAD, Lower Extremity Peripheral Arterial Disease.


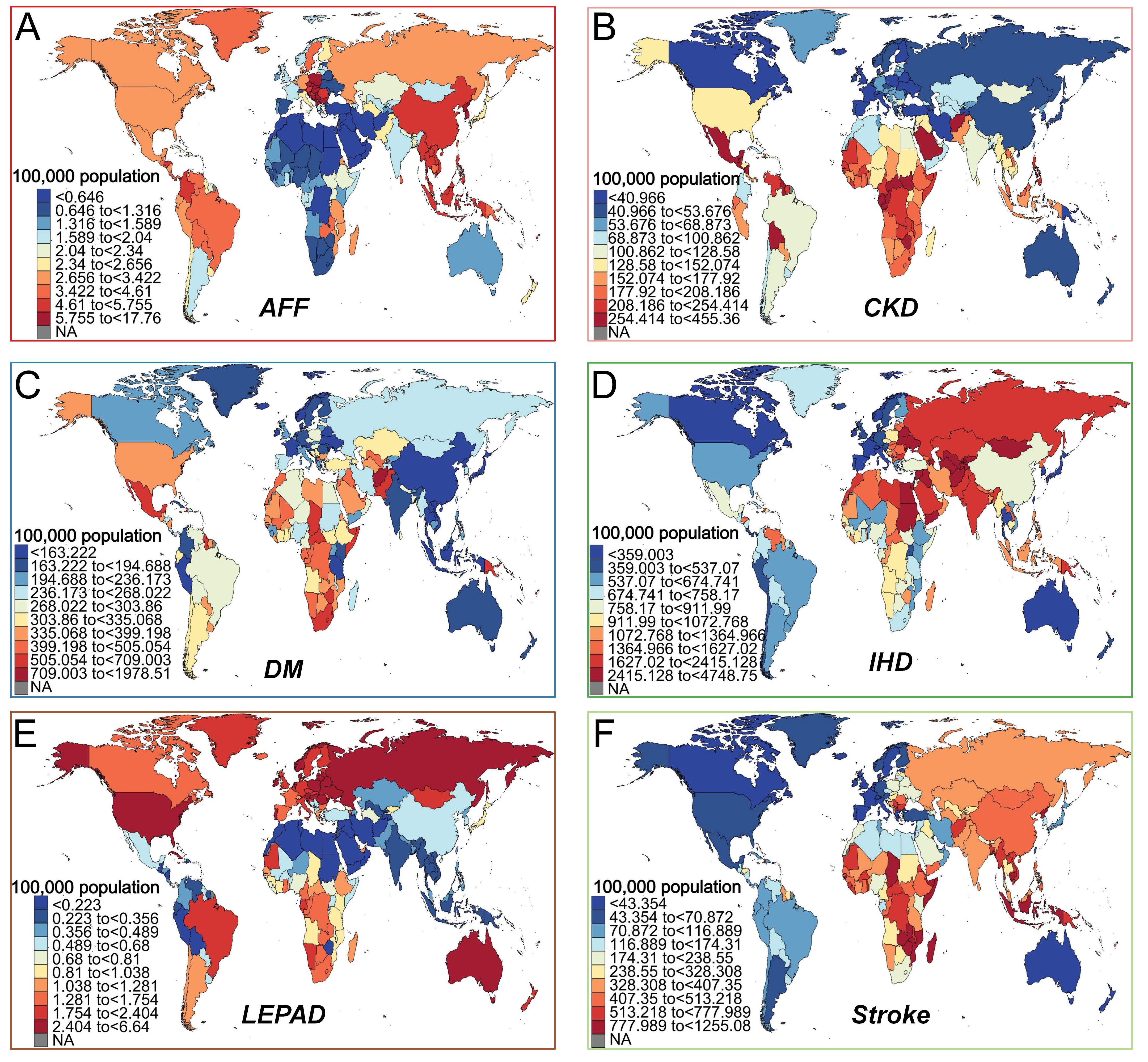

Supplement: Supplementary file 1 — Figure S1: Directed acyclic graph (DAG) for confounder selection. This DAG illustrates the assumed causal relationships between dietary nutrient intake (exposure) and CKM syndrome (outcome). Variables such as age, gender, race/ethnicity, socioeconomic status (SES), smoker, alcohol consumption, and physical activity are considered potential confounders. These confounders were identified a priori as common causes of both the exposure and the outcome and were included in the multivariable models to block backdoor paths and minimize bias in the estimation of the exposure‐outcome relationship. Figure S2: Participant selection and details of the NHANES cross‐sectional study. Among 97,687 participants from 2001 to 2020, individuals missing data on BMI, eGFR, ACR, SBP, HbA1c, TC, HDL, or key nutrient intake were excluded. A total of 48,528 participants were retained for the study. Of these, 30,207 participants with complete mortality outcome data were included in the mortality risk analysis. ACR, albumin‐to‐creatinine ratio; BMI, body mass index; eGFR, estimated glomerular filtration rate; HbA1c, hemoglobin A1c; HDL, high‐density lipoprotein; SBP, systolic blood pressure; TC, total cholesterol. Figure S3: Global distribution of ASDR for six CKM‐related diseases attributable to dietary risks. The world map illustrates the distribution of age‐standardized disability‐adjusted life years (ASDR) for six CKM‐related diseases. Darker colors indicate higher ASDR values. AFF, atrial fibrillation and flutter; CKD, chronic kidney disease; DM, diabetes mellitus; IHD, ischemic heart disease; LEPAD, lower extremity peripheral arterial disease. Figure S4: Global distribution of ASDR for six ckm‐related diseases attributable to dietary risks. The world map illustrates the distribution of age‐standardized mortality rates (ASDR) for six CKM‐related diseases. Darker colors indicate higher ASMR values. AFF, atrial fibrillation and flutter; CKD, chronic kidney disease; DM, diabetes mellitus; IH [file FSN3-14-e71747-s002.docx]
